# Supplementary material for: Vibrio cholerae Chromosome Partitioning without Polar Anchoring by HubP
Source: Genes (Basel). 2022 May 13;13(5):877. doi: 10.3390/genes13050877 (PMC9140986; doi:10.3390/genes13050877)
Supplement: Supplementary file 1 [file genes-13-00877-s001.zip › genes-1718997-Supplementary Figures.pdf]

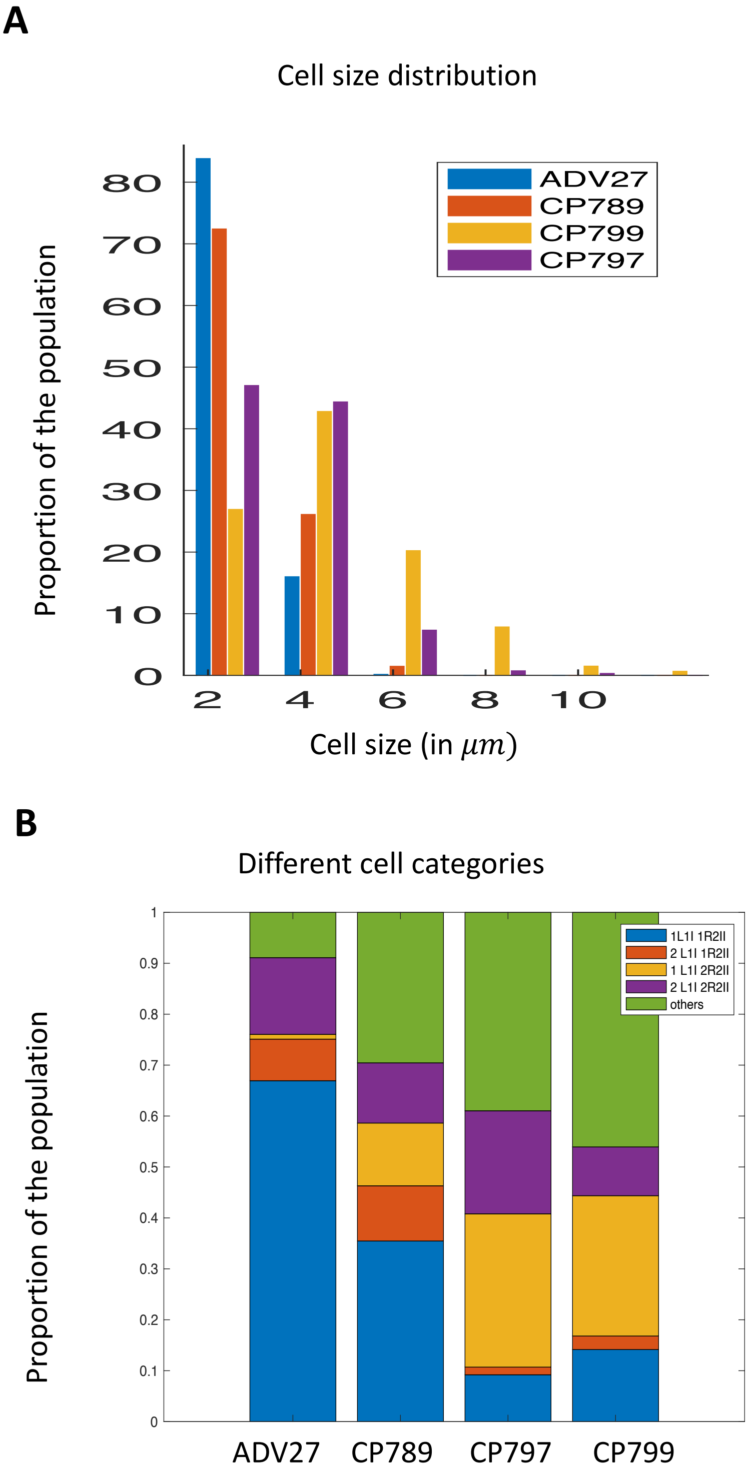

Figure S1: Characteristics of strains ADV27 (WT), CP789 ((Δ*parAB2 +ori2::2parS1)* , CP797 ($\Delta hubP \Delta$ *parAB2 +ori2::2parS1)* and CP799 ($\Delta parS1$ Δ *parAB2 +ori2::2parS1)*. **(A)** Histogram representing the proportion of cells with a length ranging within the different 2 µm size intervals, from 2 to 8 µm. **(B)** Proportion of cells ranging in different categories defined from the number of « red » and « green » foci as indicated in the figure inset.

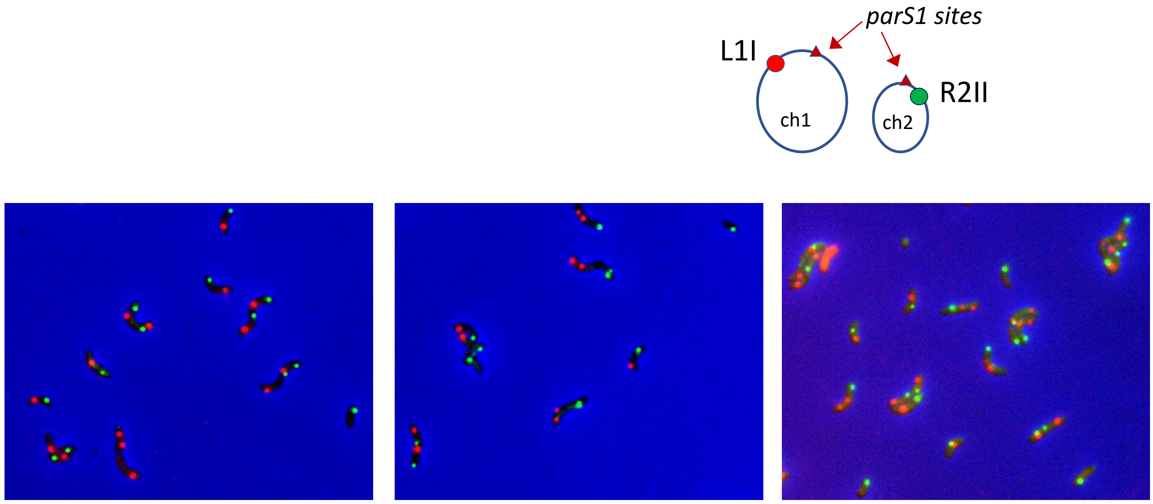

Figure. S2A: Representative Snapshots of CP789 (Δ*parAB2* +*parS1*^{ectopic} *::ori2)* containing dual labelling system (red-L1I (300kb from oriC1) and green R2II (180kb from oriC2)

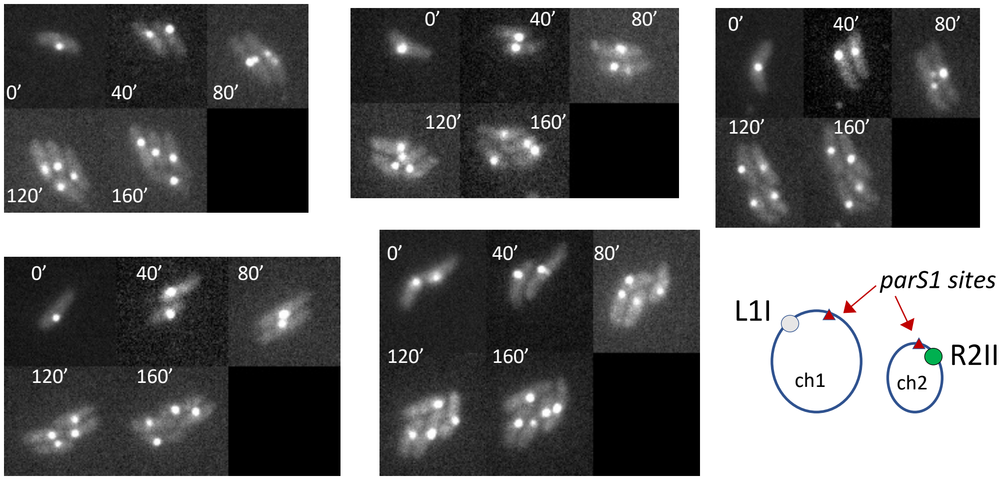

Figure. S2B: Representative timelapses of CP789 (Δ*parAB2* +*parS1*^{ectopic} *::ori2)* presenting only the GFP signal of R2II (180kb from oriC2), as the mCherry signal had photobleached.

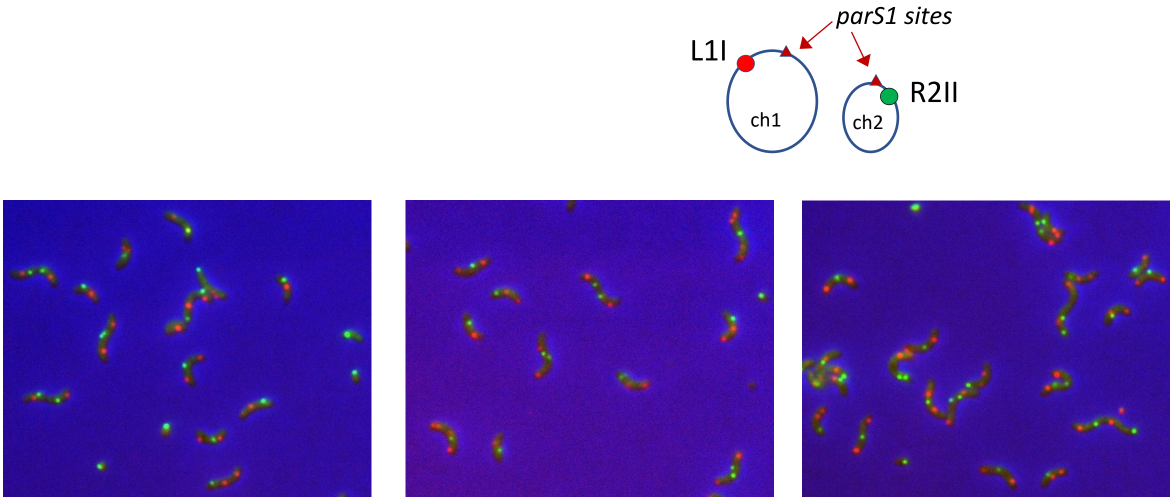

Figure. S3A: Representative Snapshots of CP797 (Δ*hubP* Δ*parAB2* +*parS1*^{ectopic}*::ori2)* containing dual labelling system (red-L1I (300kb from oriC1) and green R2II (180kb from oriC2)

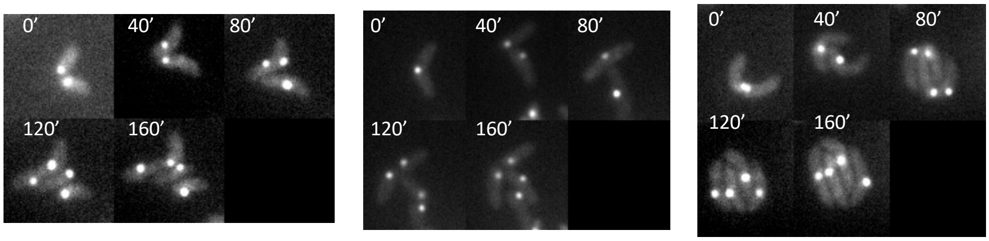

Figure. S3B: Representative timelapses of CP797 (Δ *hubP* Δ*parAB2* +*parS1*^{ectopic}*::ori2)* presenting only the GFP signal of R2II (180kb from oriC2), as the mCherry signal had photobleached.

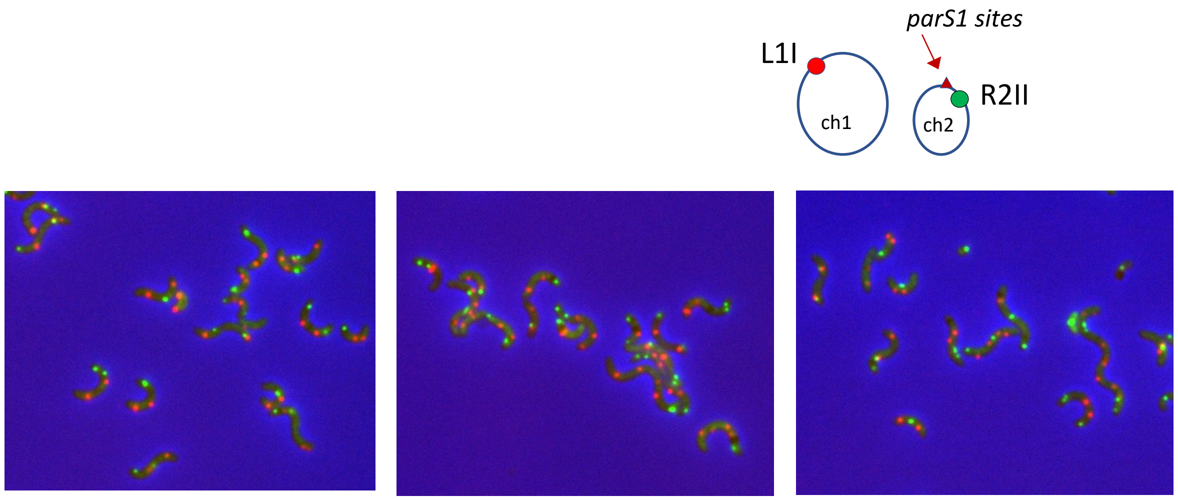

Figure. S4A: Representative Snapshots of CP799 (Δ*parS*1 Δ *parAB2* +*parS1*^{ectopic}::*ori2)* containing dual labelling system (red-L1I (300kb from oriC1) and green R2II (180kb from oriC2)

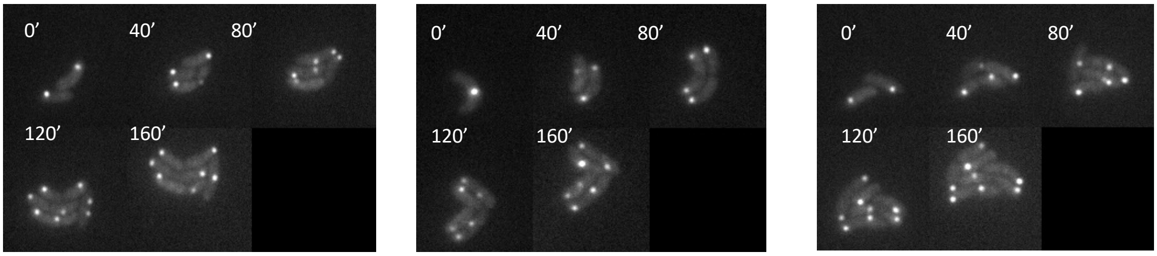

Figure. S4B: Representative timelapses of CP799 (Δ*parS*1 Δ*parAB2* +*parS1*^{ectopic}::*ori2)* presenting only the GFP signal of R2II (180kb from oriC2), as the mCherry signal had photobleached.

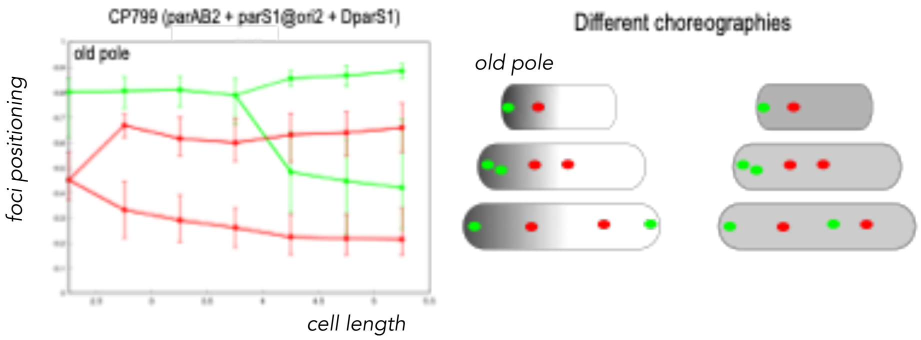

Figure S4C: Graph of reconstituted choreographies of L1I (red) and R2II (green) loci in CP799 (Δ*parS*1 Δ*parAB2* +*parS1*^{ectopic}::*ori2)*. The old pole is indicated by a gray gradient; while if no orientation biased is observed the entire cell in uniformly gray.

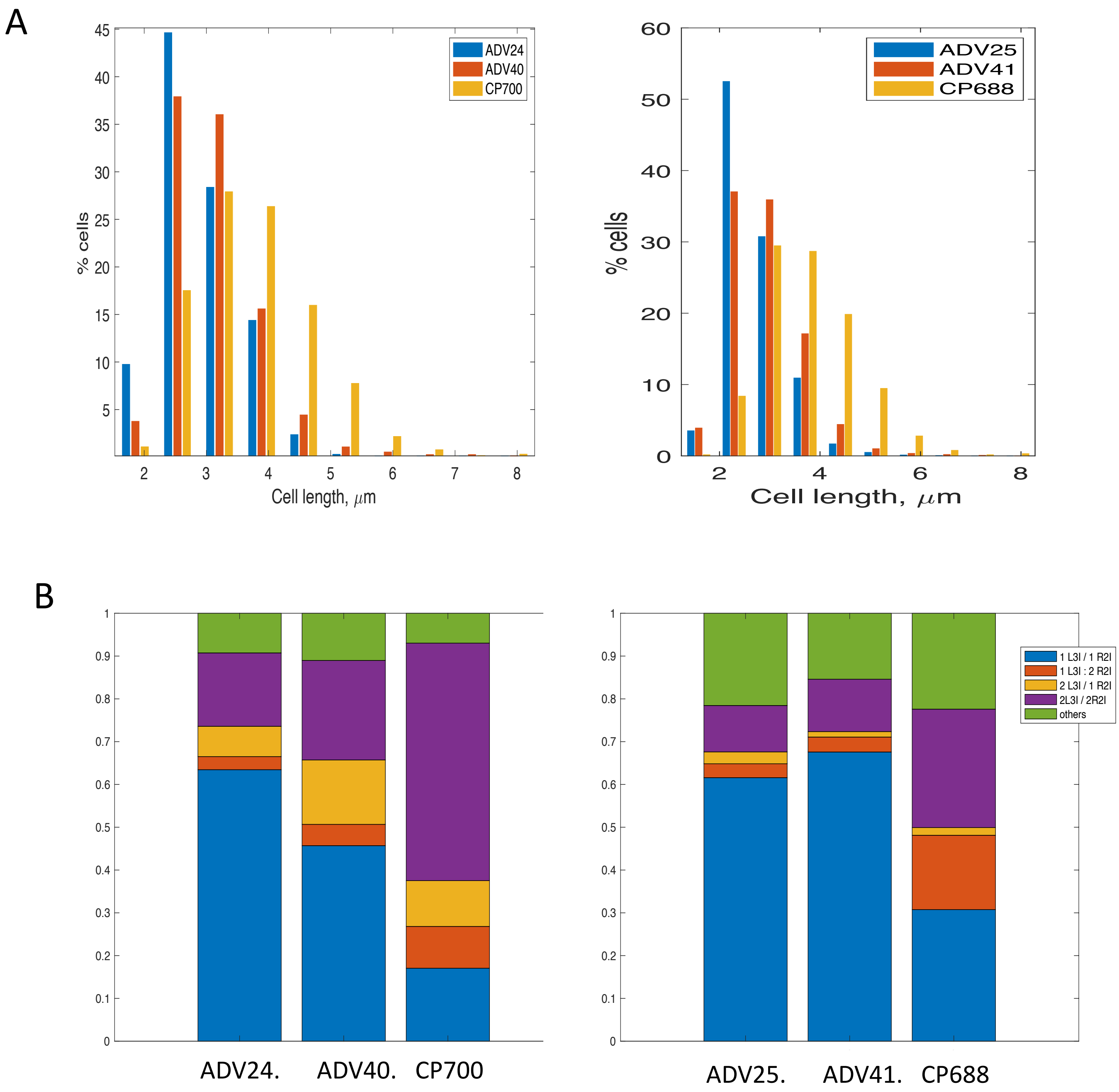

Figure S5: Characteristics of different strains: ADV24 (WT), ADV40 (*parS1*-deleted), CP700 (*hubP*-deleted) tagged at *ori1* and *L3I* and ADV25 (WT), ADV41 (*parS1*-deleted), CP688 (*hubP*-deleted) tagged at *L3I* and *R2I*. **(A)** Histogram representing the proportion of cells with a length ranging within the different 2um size intervals, from 2 to 8 µm. **(B)** Proportion of cells ranging in different categories defined from the number of « red » and « green » foci as indicated in the legend inset.

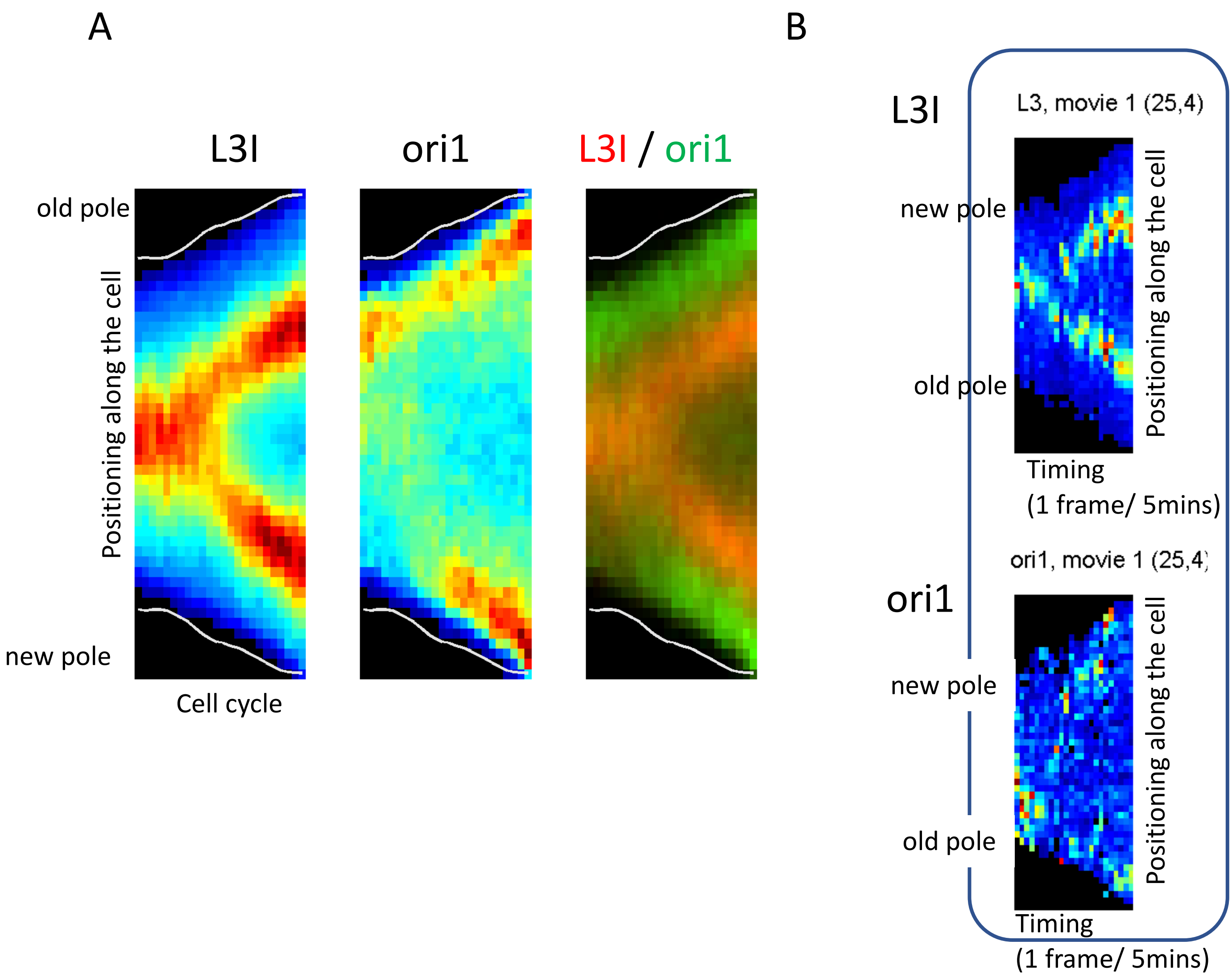

Figure. **S6:** Reconstituted choreographies of L3I and ori1 foci in CP700 from kymographs. **(A)** Compilation of the 32 lineages . Heatmap of L3I (left) , ori1 (middle) positionings and an overlay of L3I and ori1 positionings in green and red, respectively. The positionings along the cell (y axis, old pole top and new pole bottom and heatmap color corresponds to the fluorescence intensity) are in function of the progression of the cell cycle (x axis, birth to division). **(B and B1-B4)** 32 individual lineages: each composed of the two kymographs of L3I (top) and ori1 (bottom). For each kymograph, X axis corresponds to the time (1 frame/ 5mins) and Y axis corresponds to the fluorescence data projected on the long axis of the cell for each images (here, old pole bottom and new pole bottom). The intensity of the fluorescence is indicated by the heatmap color . The number of frames depends of the duration of each independent cell cycle (birth to division). The numbers in brakets allowed to identify the lineage in the timelapse.

Figure.S6B1

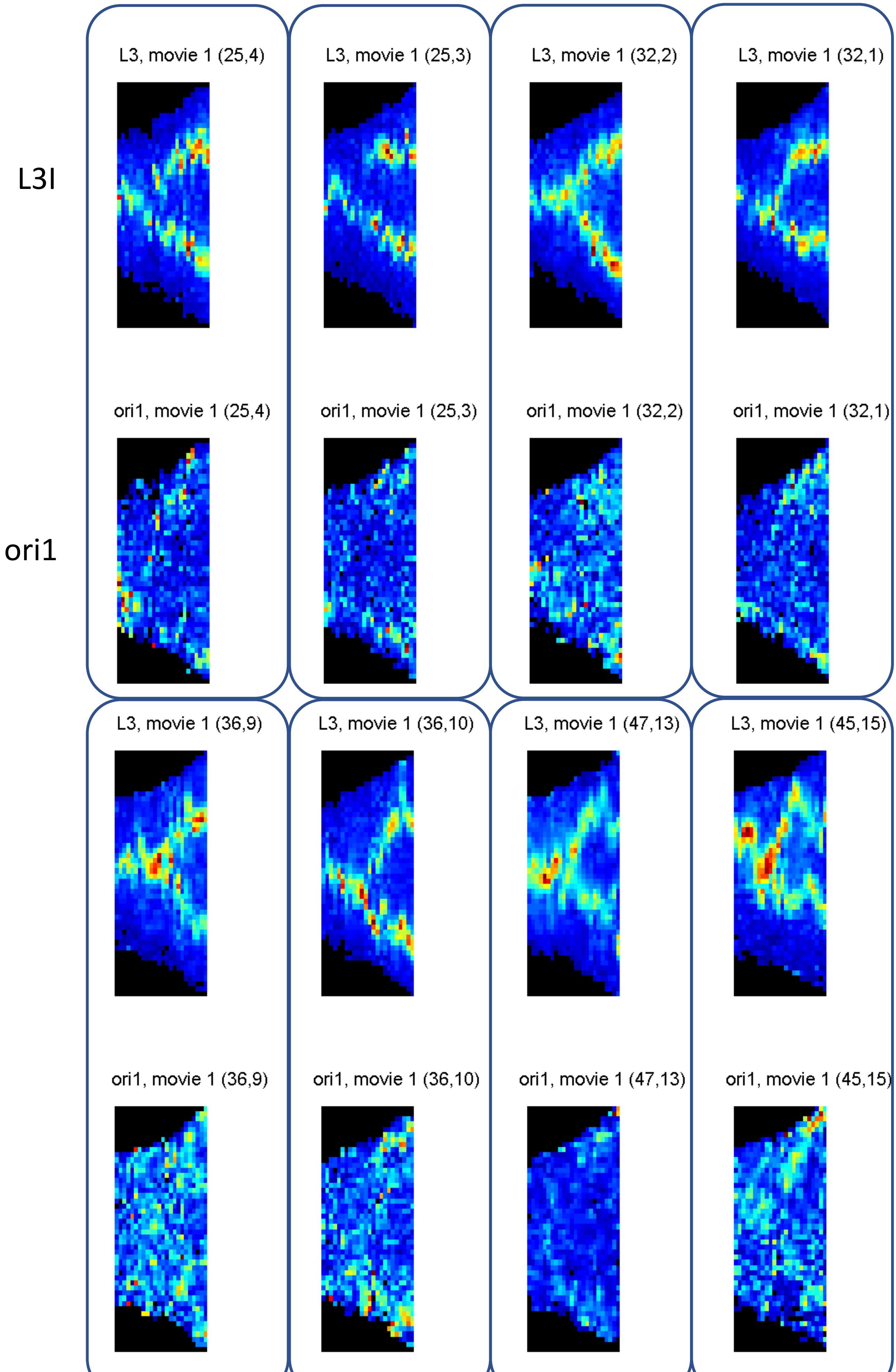

L3l
ori1
L3, movie 1 (25,4)
L3, movie 1 (25,3)
L3, movie 1 (32,2)
L3, movie 1 (32,1)
ori1, movie 1 (25,4)
ori1, movie 1 (25,3)
ori1, movie 1 (32,2)
ori1, movie 1 (32,1)
L3, movie 1 (36,9)
L3, movie 1 (36,10)
L3, movie 1 (47,13)
L3, movie 1 (45,15)
ori1, movie 1 (36,9)
ori1, movie 1 (36,10)
ori1, movie 1 (47,13)
ori1, movie 1 (45,15)

Figure.S6B2

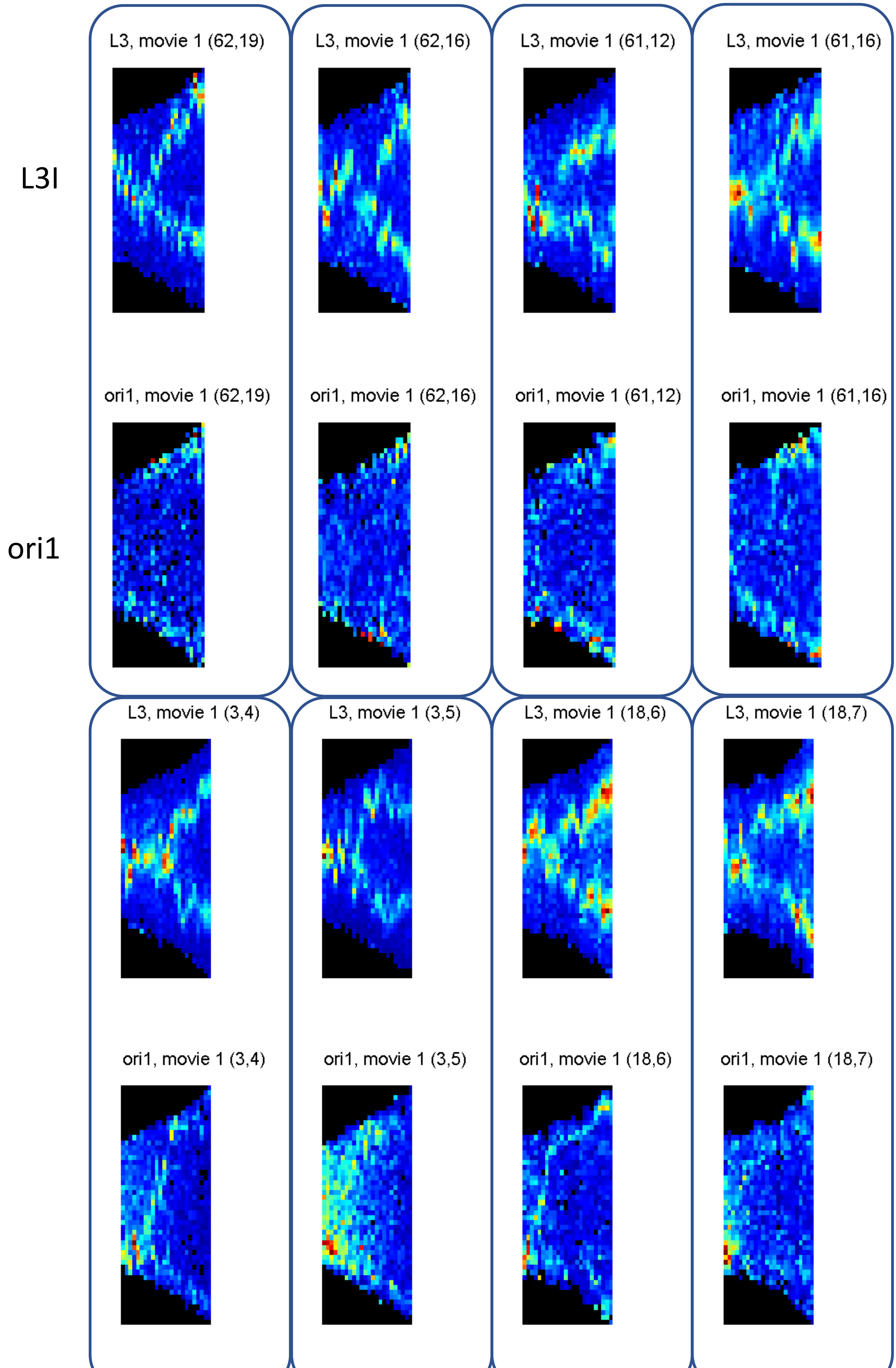
L3I
ori1
L3, movie 1 (62,19)
L3, movie 1 (62,16)
L3, movie 1 (61,12)
L3, movie 1 (61,16)
ori1, movie 1 (62,19)
ori1, movie 1 (62,16)
ori1, movie 1 (61,12)
ori1, movie 1 (61,16)
L3, movie 1 (3,4)
L3, movie 1 (3,5)
L3, movie 1 (18,6)
L3, movie 1 (18,7)
ori1, movie 1 (3,4)
ori1, movie 1 (3,5)
ori1, movie 1 (18,6)
ori1, movie 1 (18,7)

Figure.S6B3

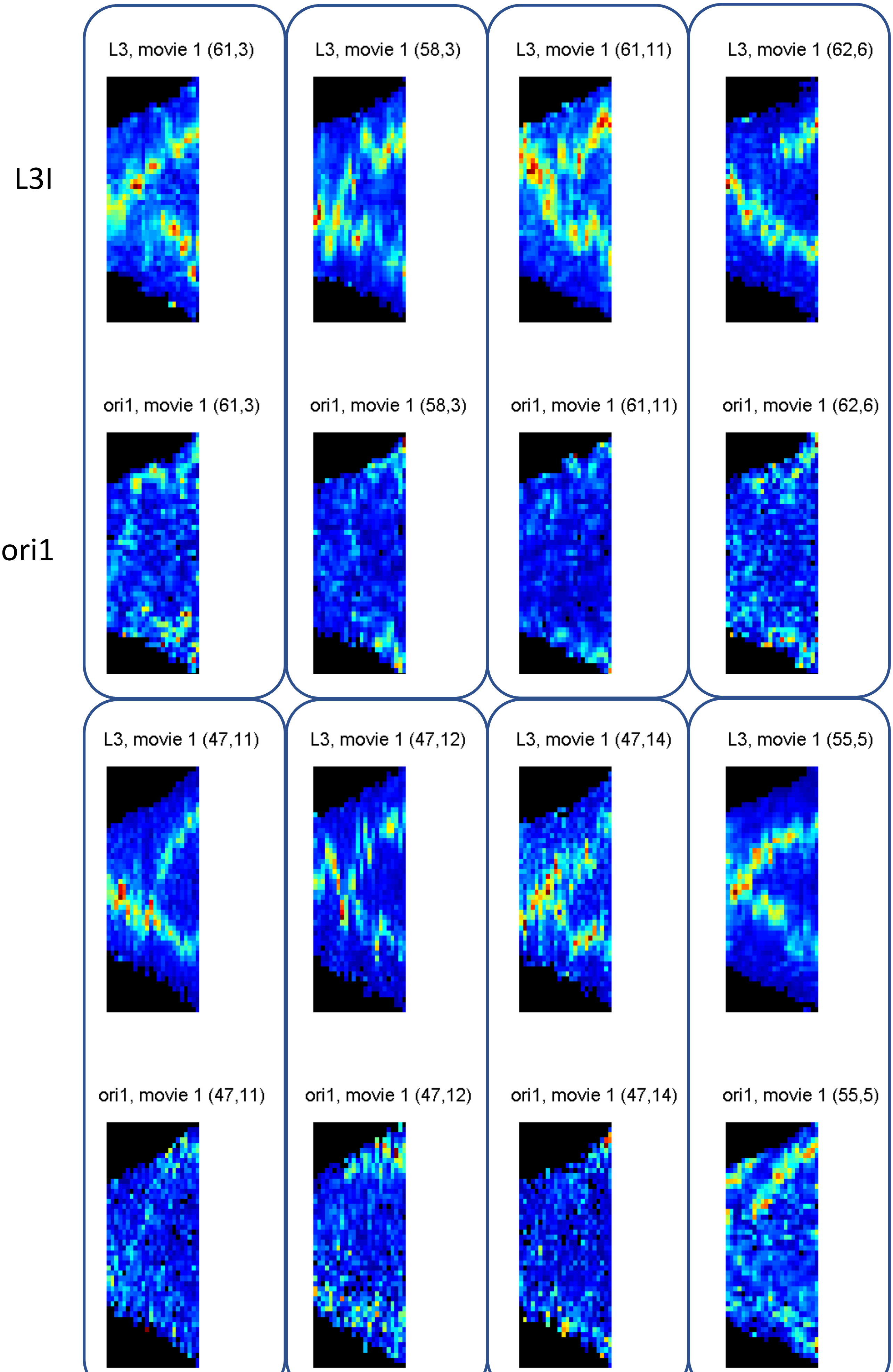
L3I
ori1
L3, movie 1 (61,3)
L3, movie 1 (58,3)
L3, movie 1 (61,11)
L3, movie 1 (62,6)
ori1, movie 1 (61,3)
ori1, movie 1 (58,3)
ori1, movie 1 (61,11)
ori1, movie 1 (62,6)
L3, movie 1 (47,11)
L3, movie 1 (47,12)
L3, movie 1 (47,14)
L3, movie 1 (55,5)
ori1, movie 1 (47,11)
ori1, movie 1 (47,12)
ori1, movie 1 (47,14)
ori1, movie 1 (55,5)

Figure.S6 B4

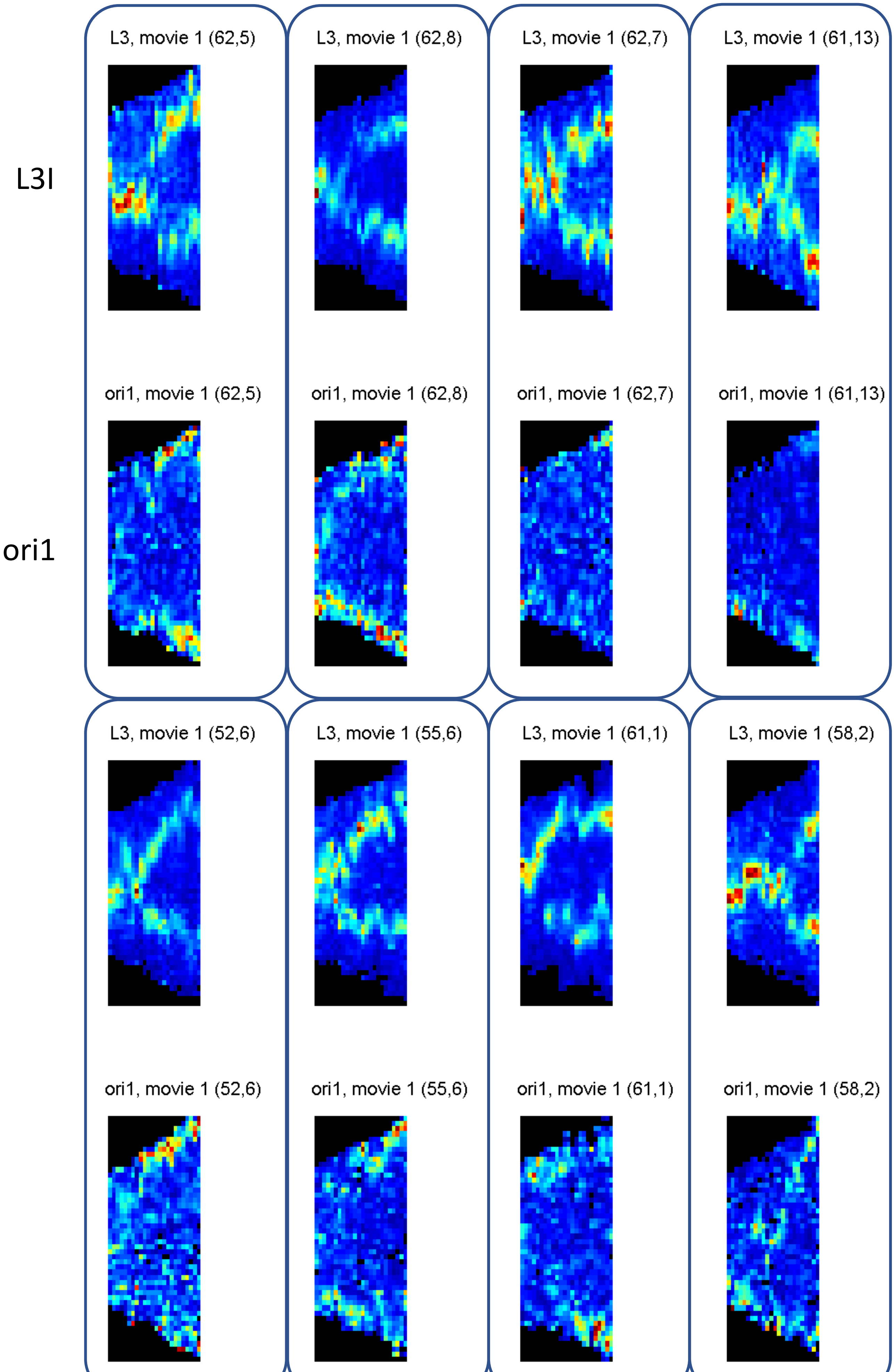

L3I
ori1
L3, movie 1 (62,5)
L3, movie 1 (62,8)
L3, movie 1 (62,7)
L3, movie 1 (61,13)
ori1, movie 1 (62,5)
ori1, movie 1 (62,8)
ori1, movie 1 (62,7)
ori1, movie 1 (61,13)
L3, movie 1 (52,6)
L3, movie 1 (55,6)
L3, movie 1 (61,1)
L3, movie 1 (58,2)
ori1, movie 1 (52,6)
ori1, movie 1 (55,6)
ori1, movie 1 (61,1)
ori1, movie 1 (58,2)

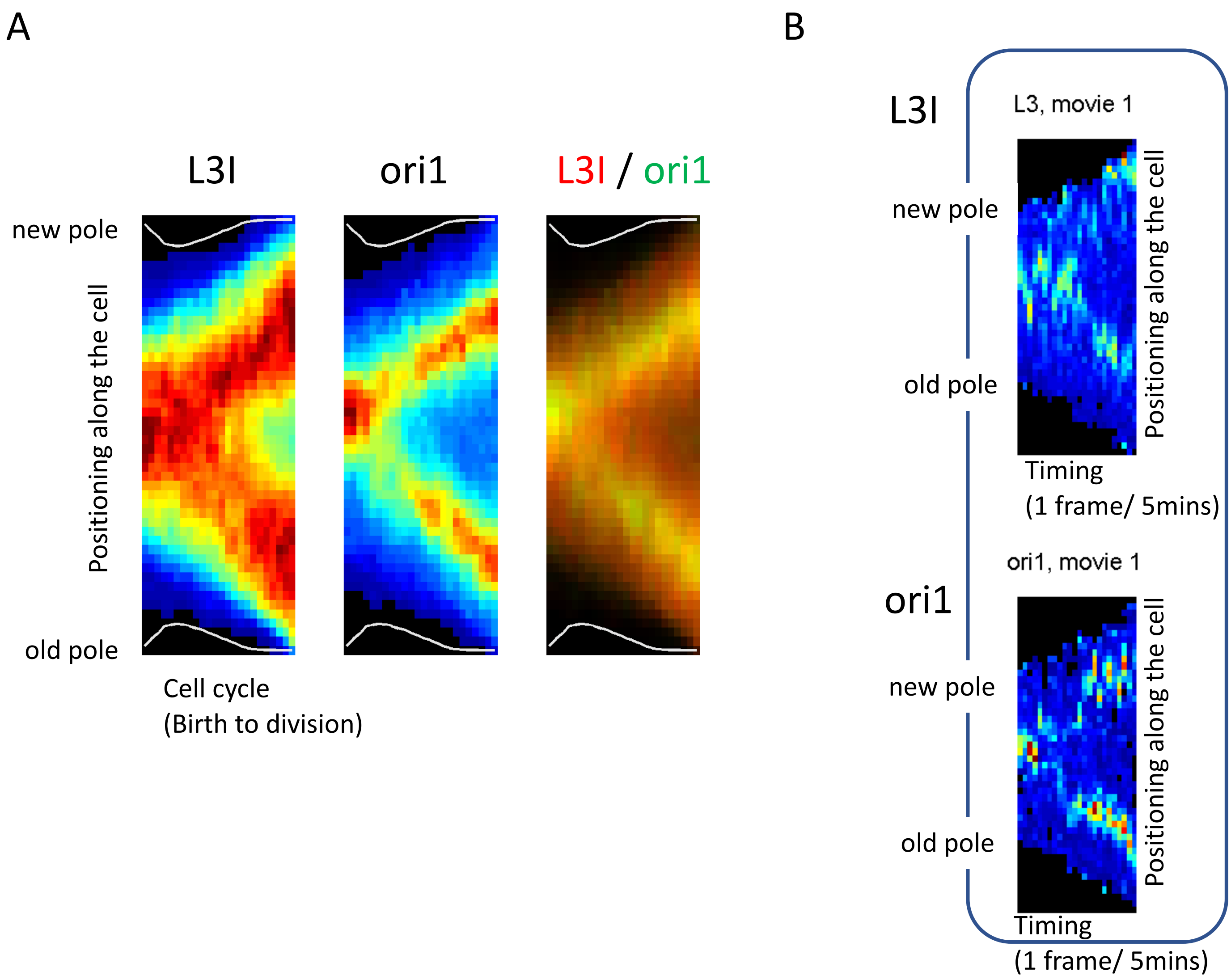

Figure. S7: Reconstituted choreographies of L3I and ori1 foci in CP700 from kymographs. **(A)** Compilation of the 56 lineages . Heatmap of L3I (left) , ori1 (middle) positionings and an overlay of L3I and ori1 positionings in green and red, respectively. The positionings along the cell (y axis, old pole top and new pole bottom) are in function of the progression of the cell cycle (x axis, birth to division and heatmap color corresponds to the fluorescence intensity). **(B and B1-B7)** 56 individual lineages: each composed of the two kymographs of L3I (top) and ori1 (bottom). For each kymograph, X axis corresponds to the time (1 frame/ 5mins) and Y axis corresponds to the fluorescence data projected on the long axis of the cell for each images (here, old pole bottom and new pole bottom). The intensity of the fluorescence is indicated by the heatmap color . The number of frames depends of the duration of each independent cell cycle (birth to division). The numbers in brakets allowed to identify the lineage in the timelapse.

Figure.S7B1

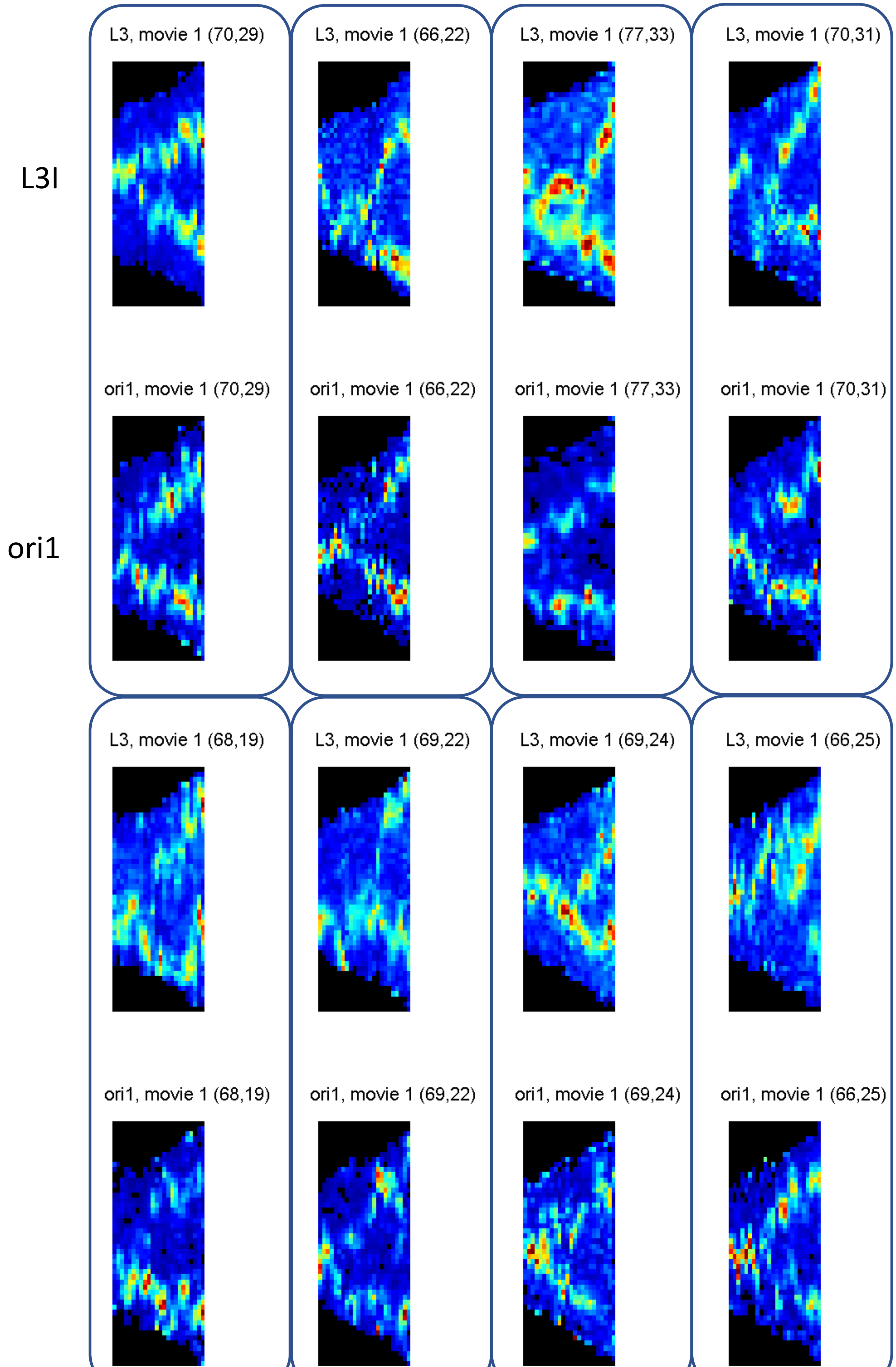

L3l
ori1
L3, movie 1 (70,29)
L3, movie 1 (66,22)
L3, movie 1 (77,33)
L3, movie 1 (70,31)
ori1, movie 1 (70,29)
ori1, movie 1 (66,22)
ori1, movie 1 (77,33)
ori1, movie 1 (70,31)
L3, movie 1 (68,19)
L3, movie 1 (69,22)
L3, movie 1 (69,24)
L3, movie 1 (66,25)
ori1, movie 1 (68,19)
ori1, movie 1 (69,22)
ori1, movie 1 (69,24)
ori1, movie 1 (66,25)

Figure.S7B2

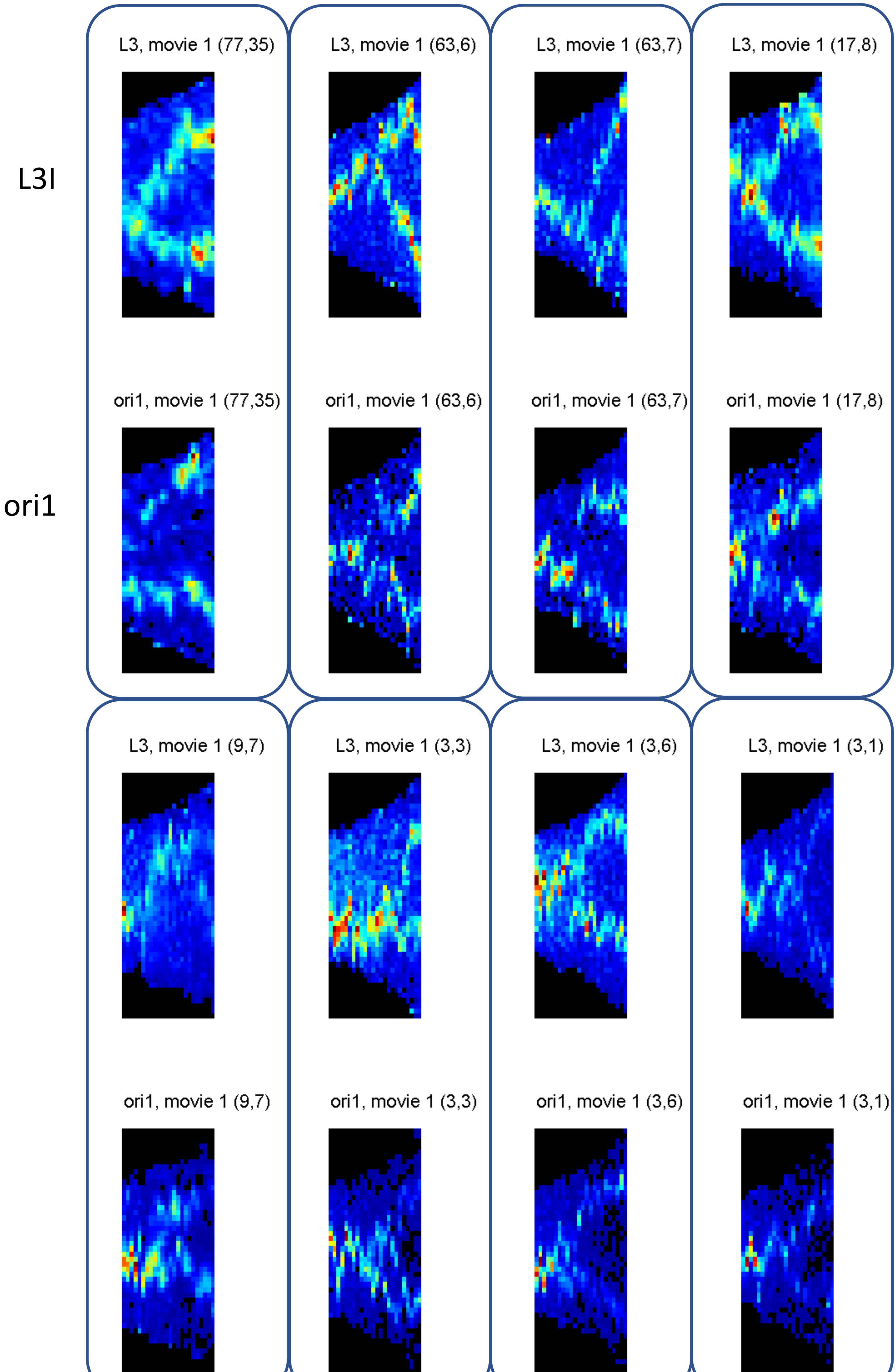
L3l
ori1
L3, movie 1 (77,35)
L3, movie 1 (63,6)
L3, movie 1 (63,7)
L3, movie 1 (17,8)
ori1, movie 1 (77,35)
ori1, movie 1 (63,6)
ori1, movie 1 (63,7)
ori1, movie 1 (17,8)
L3, movie 1 (9,7)
L3, movie 1 (3,3)
L3, movie 1 (3,6)
L3, movie 1 (3,1)
ori1, movie 1 (9,7)
ori1, movie 1 (3,3)
ori1, movie 1 (3,6)
ori1, movie 1 (3,1)

Figure.S7B3

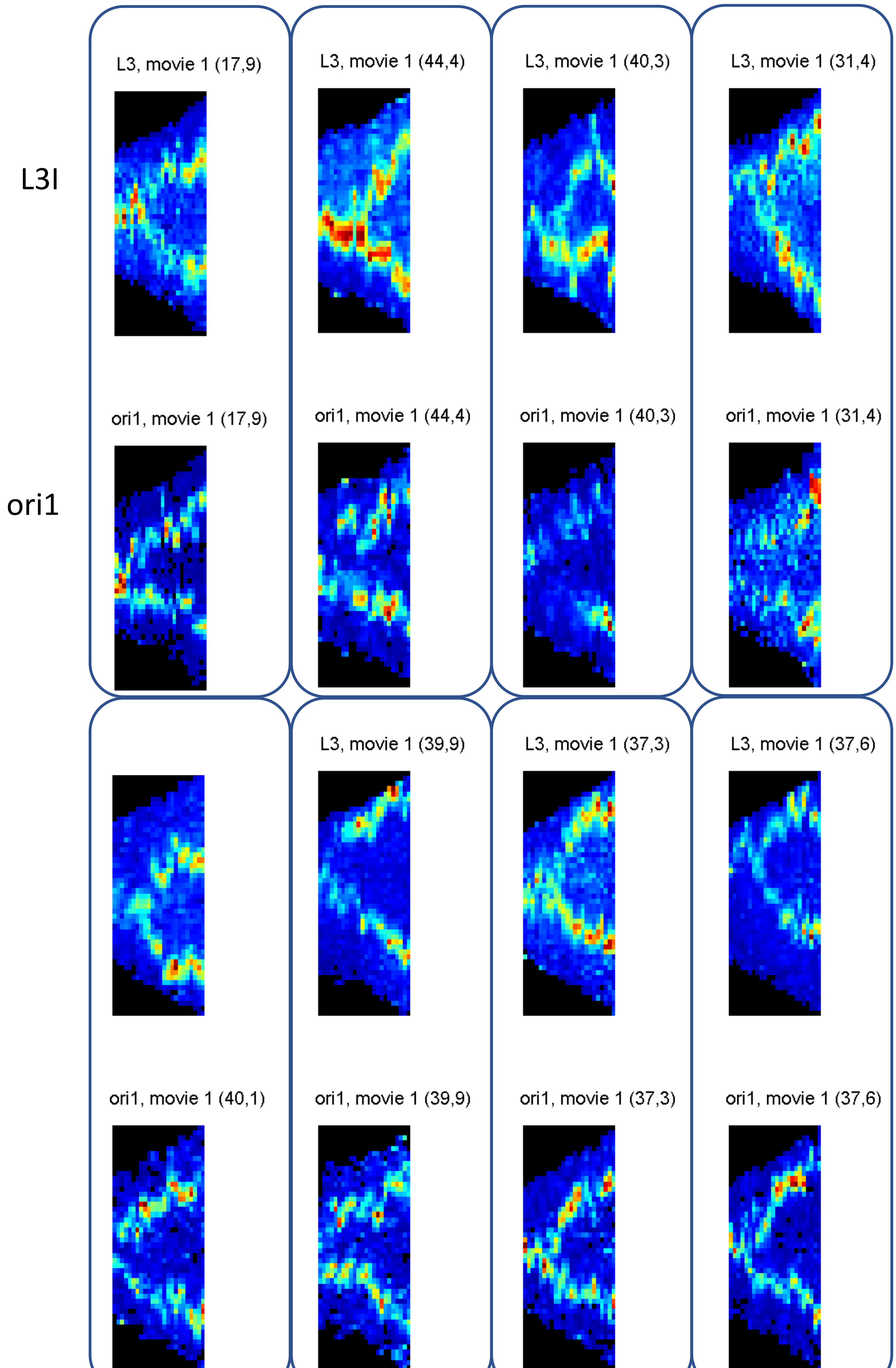
L3I
ori1
L3, movie 1 (17,9)
L3, movie 1 (44,4)
L3, movie 1 (40,3)
L3, movie 1 (31,4)
ori1, movie 1 (17,9)
ori1, movie 1 (44,4)
ori1, movie 1 (40,3)
ori1, movie 1 (31,4)
L3, movie 1 (39,9)
L3, movie 1 (37,3)
L3, movie 1 (37,6)
ori1, movie 1 (40,1)
ori1, movie 1 (39,9)
ori1, movie 1 (37,3)
ori1, movie 1 (37,6)

Figure.S7B4

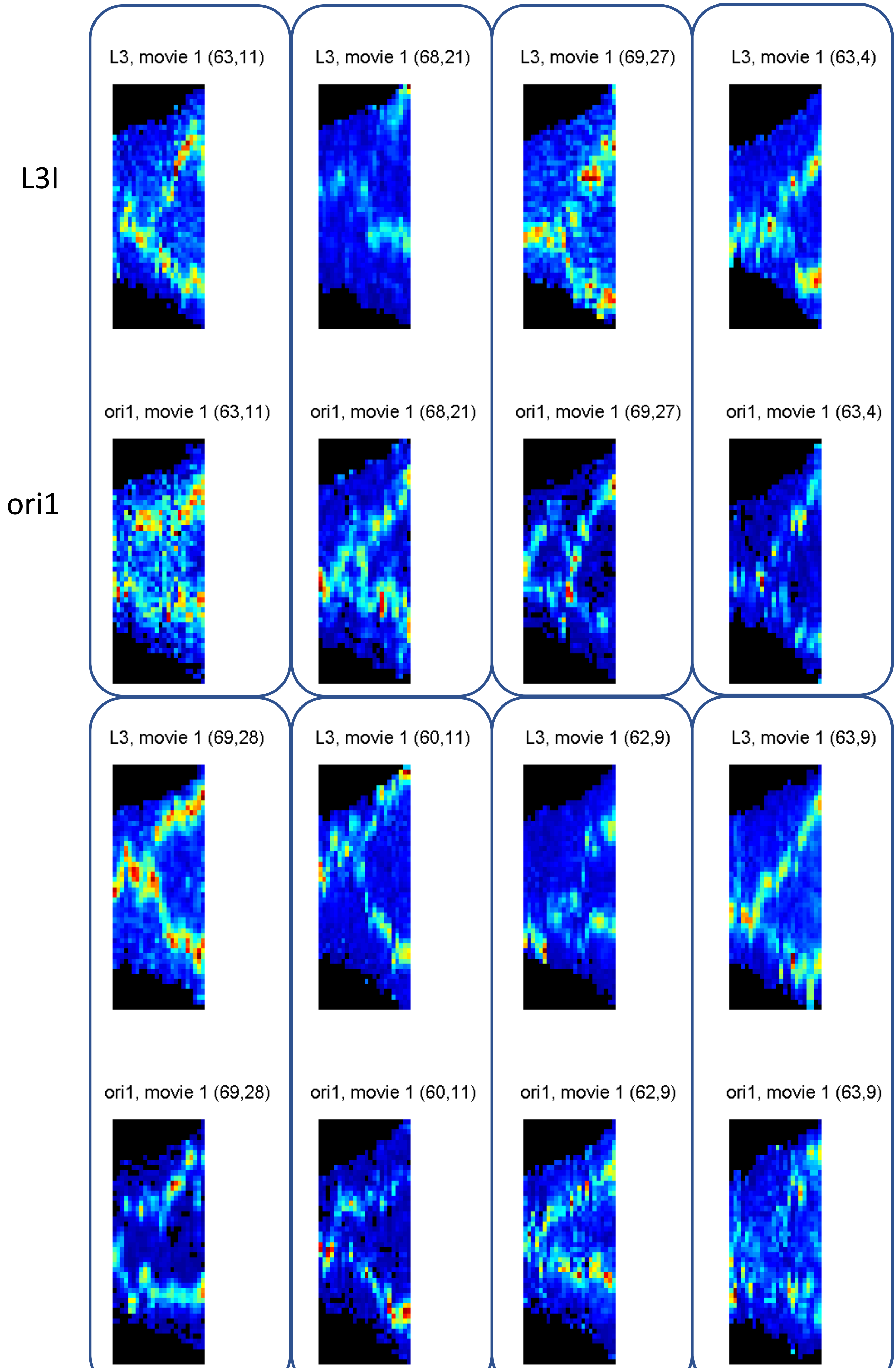
L3I
ori1
L3, movie 1 (63,11)
L3, movie 1 (68,21)
L3, movie 1 (69,27)
L3, movie 1 (63,4)
ori1, movie 1 (63,11)
ori1, movie 1 (68,21)
ori1, movie 1 (69,27)
ori1, movie 1 (63,4)
L3, movie 1 (69,28)
L3, movie 1 (60,11)
L3, movie 1 (62,9)
L3, movie 1 (63,9)
ori1, movie 1 (69,28)
ori1, movie 1 (60,11)
ori1, movie 1 (62,9)
ori1, movie 1 (63,9)

Figure.S7B5

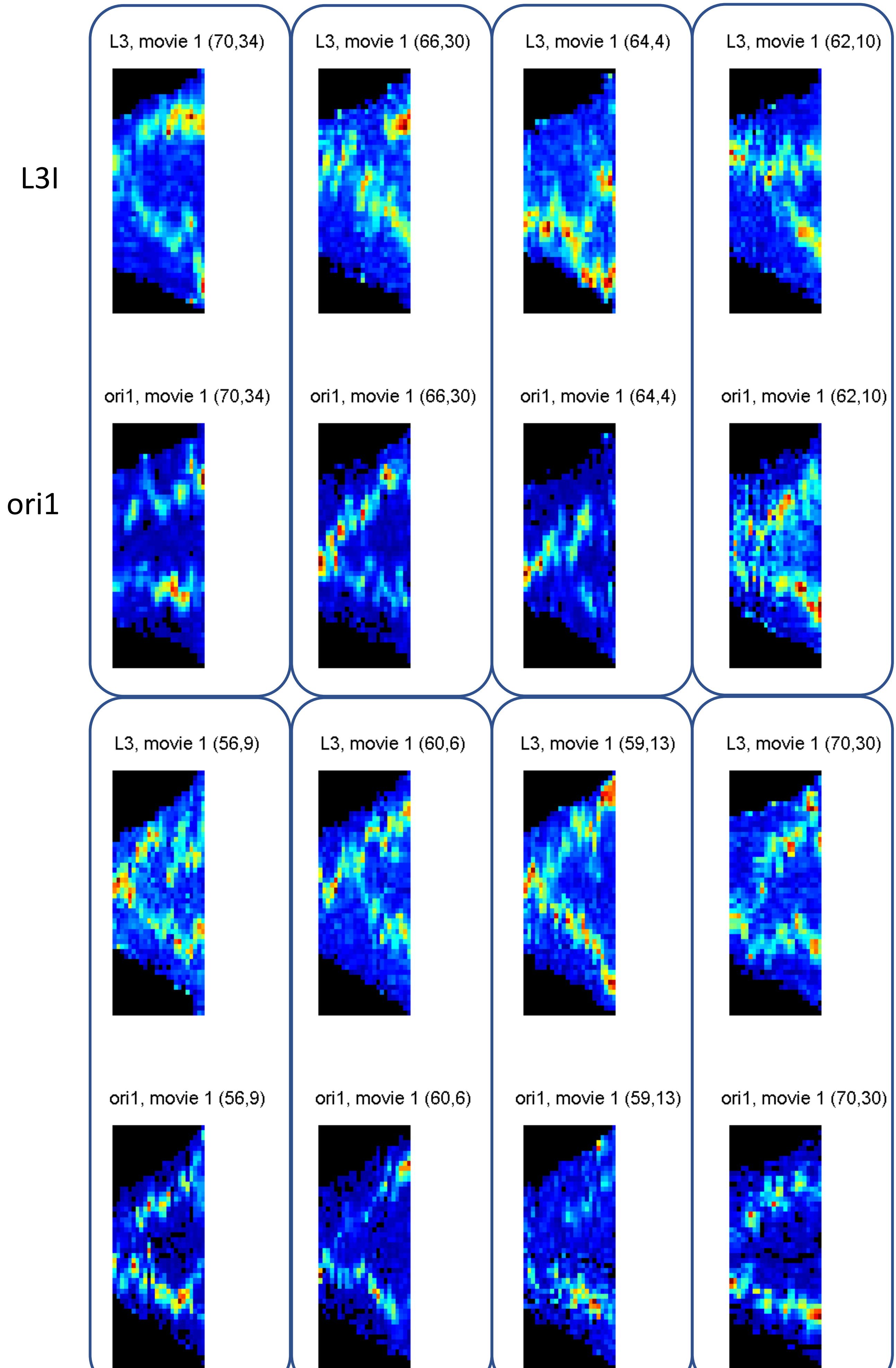
L3I
ori1
L3, movie 1 (70,34)
L3, movie 1 (66,30)
L3, movie 1 (64,4)
L3, movie 1 (62,10)
ori1, movie 1 (70,34)
ori1, movie 1 (66,30)
ori1, movie 1 (64,4)
ori1, movie 1 (62,10)
L3, movie 1 (56,9)
L3, movie 1 (60,6)
L3, movie 1 (59,13)
L3, movie 1 (70,30)
ori1, movie 1 (56,9)
ori1, movie 1 (60,6)
ori1, movie 1 (59,13)
ori1, movie 1 (70,30)

Figure.S7B6

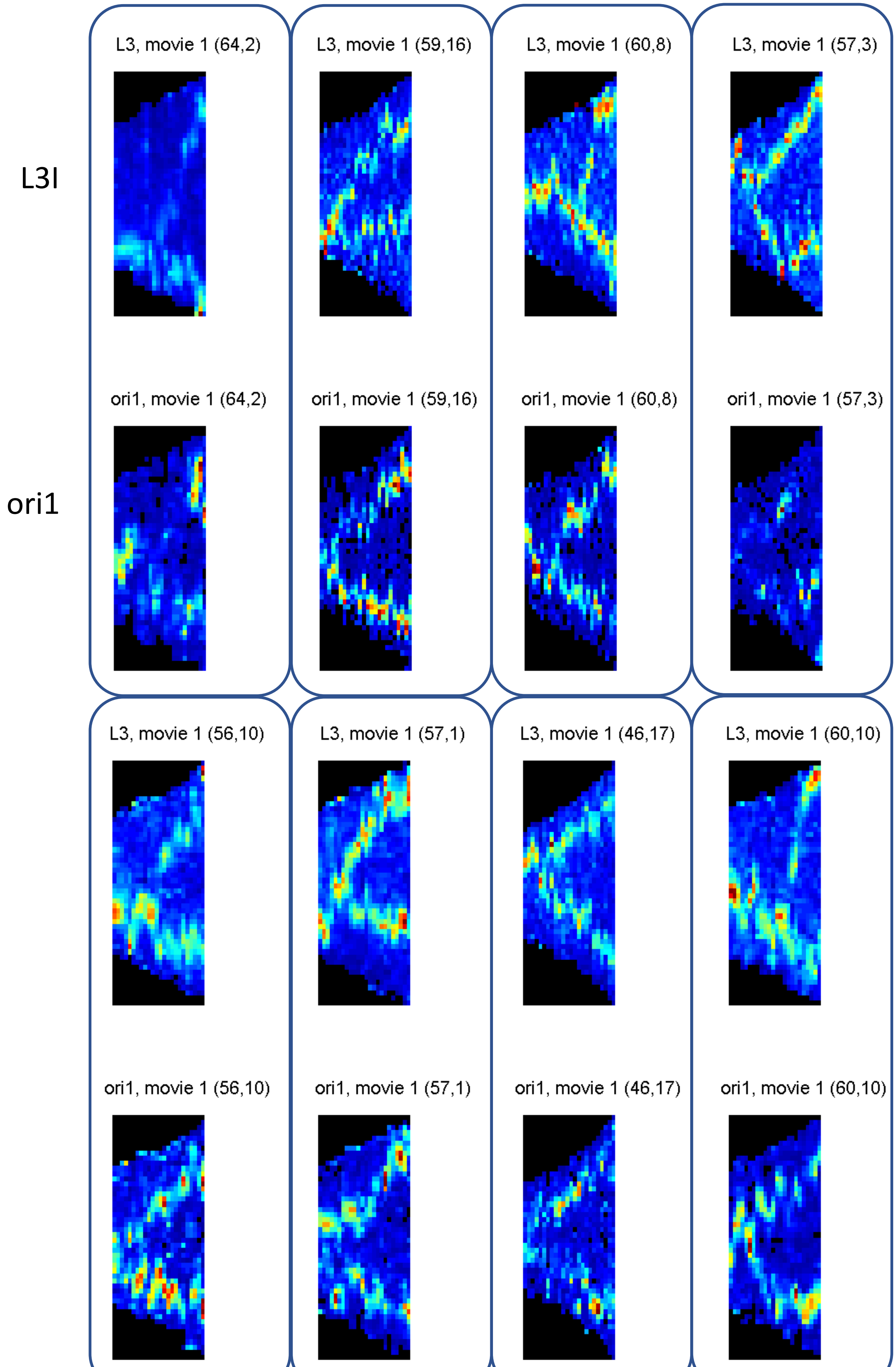

L3l
ori1
L3, movie 1 (64,2)
L3, movie 1 (59,16)
L3, movie 1 (60,8)
L3, movie 1 (57,3)
ori1, movie 1 (64,2)
ori1, movie 1 (59,16)
ori1, movie 1 (60,8)
ori1, movie 1 (57,3)
L3, movie 1 (56,10)
L3, movie 1 (57,1)
L3, movie 1 (46,17)
L3, movie 1 (60,10)
ori1, movie 1 (56,10)
ori1, movie 1 (57,1)
ori1, movie 1 (46,17)
ori1, movie 1 (60,10)

Figure.S7B7

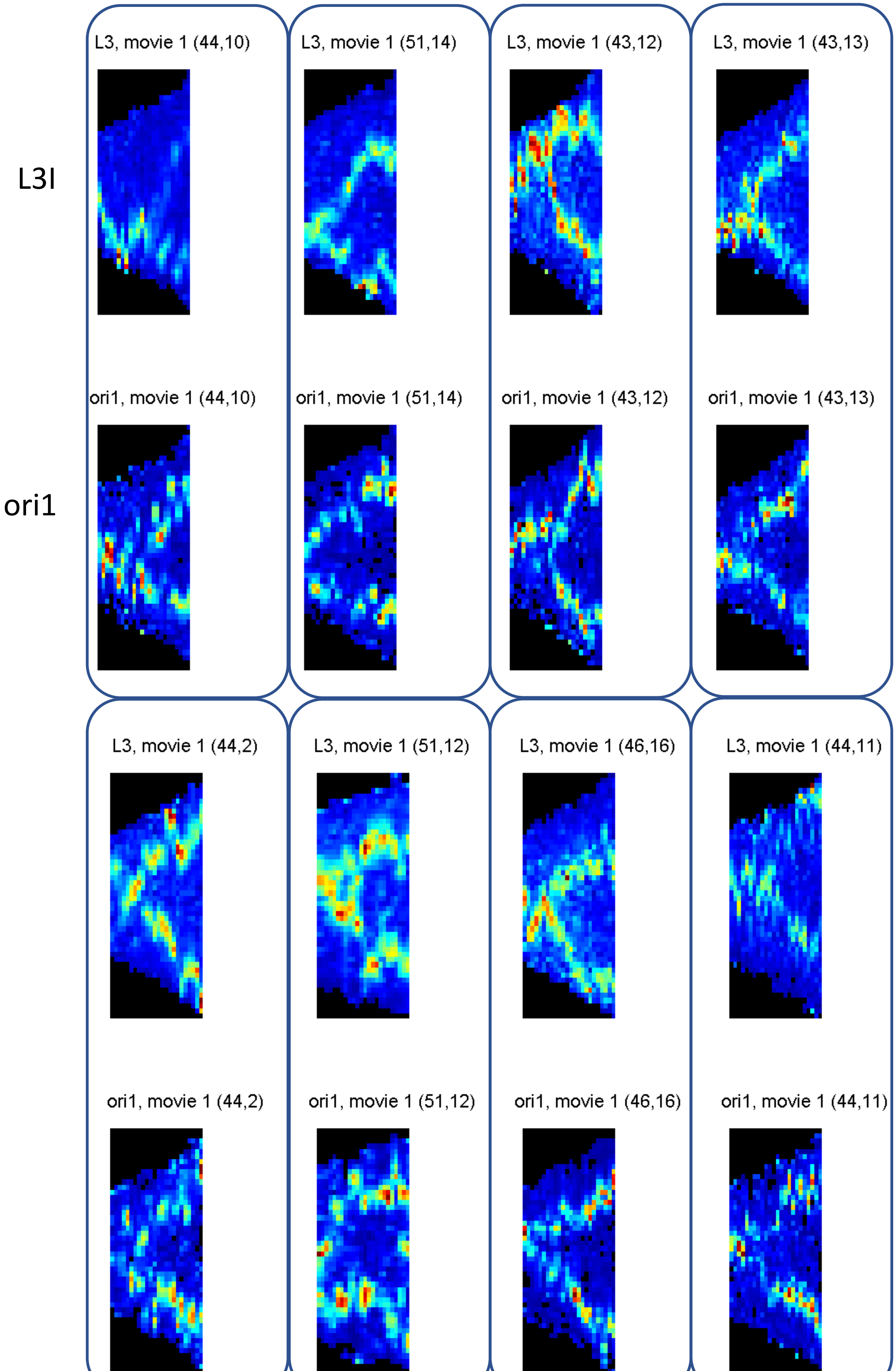
L3I
ori1
L3, movie 1 (44,10)
L3, movie 1 (51,14)
L3, movie 1 (43,12)
L3, movie 1 (43,13)
ori1, movie 1 (44,10)
ori1, movie 1 (51,14)
ori1, movie 1 (43,12)
ori1, movie 1 (43,13)
L3, movie 1 (44,2)
L3, movie 1 (51,12)
L3, movie 1 (46,16)
L3, movie 1 (44,11)
ori1, movie 1 (44,2)
ori1, movie 1 (51,12)
ori1, movie 1 (46,16)
ori1, movie 1 (44,11)
