# Supplementary material for: Vibrio cholerae Chromosome Partitioning without Polar Anchoring by HubP
Source: Genes (Basel). 2022 May 13;13(5):877. doi: 10.3390/genes13050877 (PMC9140986; doi:10.3390/genes13050877)
Supplement: Supplementary file 1 [file genes-13-00877-s001.zip › genes-1718997-Supplementary Tables.pdf]

Table S1. List of strains used

Names	Relevant genotype	genotype	plasmid used or reference
**ADV4**	WT	N16961 attB Tn7::hapR+,<br>lacZ ::(LacI-mCherry , yGFP-parBT1)	[8]
**ADV22**	WT	ADV4 parST1 -cat @ L3I : 2,31 Mb ,<br>LacOarray -Kan @ L1I : 2,66 Mb	[8]
**ADV24**	WT	ADV4 parST1 -cat @ L3I : 2,31 Mb ,<br>LacOarray -Kan @ ori1 : 0,05 Mb	[8]
**ADV25**	WT	ADV4 parST1 -cat @ L3I : 2,31 Mb ,<br>LacOarray -Kan @ R2I : 0,85 Mb	[8]
**ADV27**	WT	ADV4, lacOarray -Kan @ ori2 : 1,05 Mb<br>parST1 -cat @ R3II : 0,312 Mb	[8]
**ADV40**	△3parS1	ADV24 parS1::zeoR	pPOS188
**ADV41**	△3parS1	ADV25 parS1::zeoR	pPOS188
**ADV80**	WT	ADV4, parST1 -cat @ R1II : 0,064 Mb	pAD39
**CP578**	WT	ADV80, tet-parS1-arr2-parS1-tet @ R1II : 0,064 Mb	pPOS184
**CP768**	△parAB2	CP578,LacOarray -Kan @ L1I : 2,66 Mb	pPOS209
**CP688**	△hubP	ADV25 hubP::zeoR	pEG266
**CP700**	△hubP	ADV24 hubP::zeoR	pEG266
**CP770**	△parAB2	ADV80, parAB2::aad1	pPOS209
**CP789**	△parAB2,<br>ori2 ::2parS1	CP770, tet-parS1-arr2-parS1-tet @ R1II : 0,064 Mb,	pPOS184
**CP797**	△parAB2, ori2 ::2parS1I,<br>△hubP	CP789, hubP::zeoR	pEG266
**CP799**	△parAB2, ori2 ::2parS1,<br>△3parS1	CP789, 3parS1::zeoR	pPOS188

Tables S2. List of Plasmids used

**Plasmid**	**Description**
**Reference / construction***	
**pXX705**	Mini-F plasmid vector; Fori, Amp-R
**[35]**	
**pYB145**	pXX705 parAB2 parS2
**[32]**	
**pYB153**	TOPO-parAB1
**parAB1 genes was amplified with YPR254 x YPR255 then cloned into pCR Blunt II-TOPO vector (ThermoFisher)**	
**pYB164**	pXX705 parS1
**parS1 dsDNA was generated by annealing YPR179 and YPR180, and cloned into the EcoRI site of pXX705.**	
**pYB167**	pXX705 parAB1 parS1
**parAB1 fragment was obtained by BamHI digestion of pYB153 and cloned into pYB164**	
**pYB172**	pXX705 parAB1
**parAB1 fragment was obtained by BamHI digestion of pYB153 and cloned into pXX705**	
**pGD162**	; sacB ; ori R6K ; Tet'-zeo-'Tet cassette ; CmR, ZeoR
**[8]**	
**pAD19**	sacB ; ori R6K ; YGFP-ParBT1 and LacI-mcherry between Up- and Down-stream regions of V.cholerae LacZ gene ; CmR
**[8]**	
**pAD20**	sacB ; ori R6K ; LacO array + KanR between TetR homologies ; CmR, Kan
**[8]**	
**pAD22**	integration-excision vector ; sacB ; Tet-Zeo-Tet cassette between homologies around LII ; CmR, ZeoR
**[8]**	
**pAD39**	sacB ; ori R6K ; Tet'-parST1- FRT-CmR-FRT-'Tet ; CmR
**[8]**	
**pPOS188**	sacB ; ori R6K ; Δ3parSI :: zeoR
**See Mat. & Met.**	
**pPOS184**	sacB ; ori R6K ; parSI-FRT-Rif-FRT-parSI between TetR homologies ; CmR, Rif
**[8]**	
**pPOS209**	sacB ; ori R6K ; ΔparAB2 :: specR
**See Mat. & Met.**	
**pEG266**	sacB ; ori R6K ; ΔhubP :: zeoR
**[58]**	

Table S3. Oligo DNAs used in plasmids construction

Name	Sequence (5'-3')
**YPR179**	AATTCCGTTTCACGTGAAACAG
**YPR180**	AATTCTGTTTCACGTGAAACGG
**YPR254**	CGGGATCCAATCACCACAGCTCGAAC
**YPR255**	CGGGATCCGCGGCCGCAGTGGAAGAGTTTGATCCTG
**parAB2 DW SacI**	CCgagctcGCAAAGGATTGGTACTTTCTGCAG
**parAB2 DW XhoI**	GCctcgagGTCAACTCAAATAACAAAGCCC
**parAB2 UP BamHI**	GCggatccTTTCCGTTATTAGGGCTTTAAAC
**parAB2 UP xbaI**	CCtctagaGCAATGAATTTACATGCTTCCGCG
**parS1 UP1 xbaI**	GGCTtctagaCATGAGGCGCCATTCTATACCC
**parS1DW1 BamHI**	CTGCggatccATGCACAAACCATCTCCAATCG
**parS1 UP2 xhoI**	GCGTctcgagCAGTTATTTGCTGGCCCATTAG
**parS1 DW2 sacI**	GGCTgagctcCAGAATGCGAGCCATTTCATAG
